# A Multidisciplinary Approach to Diagnosis and Management of Adult Plumbism Complicated by Cerebral Herniation: A Case Report

**DOI:** 10.7759/cureus.96602

**Published:** 2025-11-11

**Authors:** Sukhshant Atti, Felicia Hataway, William Rushton, Somnath Das, Benjamin Von Schweinitz

**Affiliations:** 1 Emergency Medicine, University of Alabama at Birmingham School of Medicine, Birmingham, USA; 2 Neurology, University of Alabama at Birmingham School of Medicine, Birmingham, USA; 3 Neurological Surgery, University of Alabama at Birmingham School of Medicine, Birmingham, USA

**Keywords:** brain tissue herniation, lead chelation, lead encephalopathy, lead toxicities, poison, unintentional weight loss

## Abstract

Lead encephalopathy is a rare diagnosis, and its presentation can be challenging to identify and manage. We present a case of a 22-year-old female diagnosed with acute lead encephalopathy. The patient presented to the Emergency Department with a four-month history of non-specific, vague neurological and gastrointestinal complaints. During the initial evaluation, a broad differential diagnosis required inpatient hospitalization for further workup. While hospitalized, the patient developed seizures and encephalopathy followed by cerebral herniation requiring extraventricular drain (EVD) placement and intracranial pressure (ICP) management. Radiopaque material on abdominal radiography and lab work raised concern for possible heavy metal ingestion, specifically lead. Concern arose for intentional spousal poisoning. Treatment with chelation therapy using dimercaptosuccinic acid (DMSA) and British anti-lewisite (BAL) was initiated along with whole bowel irrigation and endoscopic removal of metallic material. After resolution of her critical illness and an additional two rounds of chelation therapy, the patient has regained her baseline health. Managing lead encephalopathy poses significant challenges while concurrently managing elevated ICPs. A patient with severe lead encephalopathy requires collaboration of care across multiple disciplines.

## Introduction

Lead toxicity affects multiple organ systems, including the central nervous, hematopoietic, skeletal, cardiovascular, renal, and reproductive systems. Diagnosis is often delayed due to the nonspecific nature of early symptoms, which may include abdominal pain, anorexia, nausea, vomiting, and constipation. Neurotoxicity, particularly lead encephalopathy, represents the most severe form of lead poisoning. Lead mimics divalent calcium cations, allowing it to cross the blood-brain barrier and exert toxic effects within the central nervous system. These effects include disruption of mitochondrial function through mitochondrial transition pore dysfunction, which leads to cellular apoptosis and cerebral edema. In the existing literature, lead encephalopathy is predominantly described in pediatric populations. Adult cases are rare. Children’s developing brain and nervous systems, along with a relatively immature blood-brain barrier, render them more susceptible to elevated blood lead levels (BLLs) and the development of encephalopathy [1]. We report a rare case of severe lead encephalopathy in a 22-year-old woman, presenting with cerebral edema and herniation. Management required a multidisciplinary approach, including cerebrospinal fluid (CSF) diversion and advanced intracranial pressure (ICP) control.

## Case presentation

A 22-year-old, previously healthy female presented to an emergency department (ED) with a four-month history of vague complaints, including vomiting, constipation, abdominal pain, and anorexia with an unintentional weight loss of 18 kg. She reported syncope and one episode of transient left-sided hemiplegia in the preceding week. In the ED, vital signs were normal, and she appeared pale and emaciated. Ancillary studies showed normocytic anemia (hemoglobin 7.9 gm/dL, MCV 83 fL) and a blood smear with mild anisopoikilocytosis and rare schistocytes. While in the ED, the patient suffered a generalized tonic-clonic seizure, which was aborted with lorazepam 2 mg intravenous (IV). A non-contrast head computed tomography (CT) scan revealed a benign cystic lesion in the left temporal lobe.

**Figure 1:**
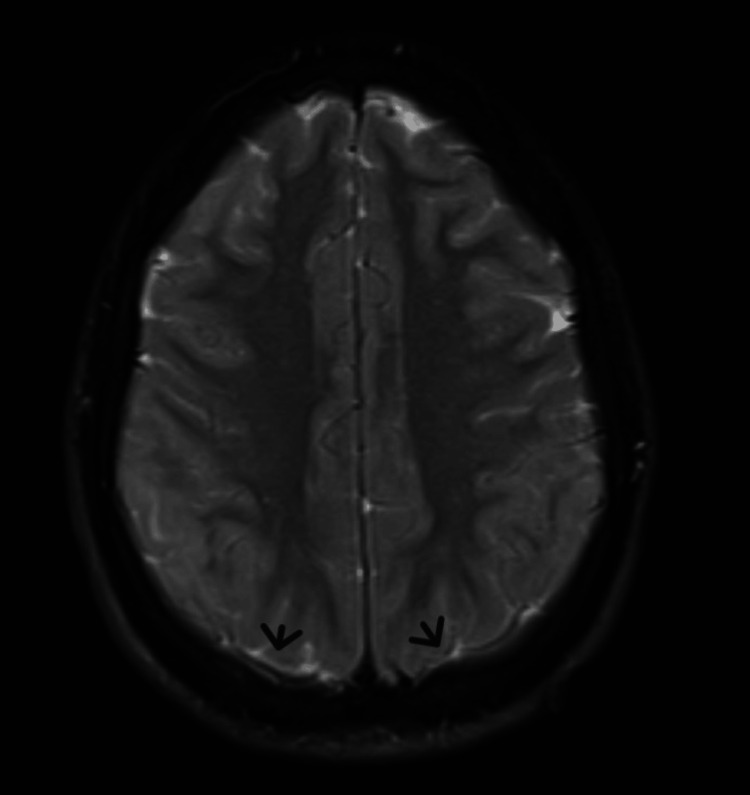
Axial MRI of the brain The scan image is demonstrating mild leptomeningeal enhancement (black arrows).

The patient remained postictal and soon developed a temperature of 38.7°C. She became intermittently somnolent, aphasic, and agitated. On initial neurological examination, she was stuporous, with roving eye movements to the left, and nuchal rigidity. The patient’s EEG demonstrated global slowing and one focal seizure. Levetiracetam 500 mg IV every eight hours and antimicrobial therapy (acyclovir 450 mg IV every eight hours, ceftriaxone 2 gm IV 12 hours) were empirically administered. Afterwards, Magnetic resonance imaging (MRI) of the brain revealed subtle FLAIR hyperintensity and mild leptomeningeal enhancement without cerebral herniation, with recommendations for lumbar puncture (LP) to rule out meningitis (Figure 1). The LP demonstrated a strikingly elevated opening pressure of >55 cm H2O with basic studies showing: glucose 80 mg/dL, protein 10 mg/dL, RBC 0, TNC 8, 33% macrophages, and 31% lymphocytes. Table 1 demonstrates an overview of the patient's pertinent laboratory findings.

**Table 1:** Serum and urine laboratory values WBC: White blood cells, HGB: Hemoglobin, HCT: Hematocrit, MCV: Mean corpuscular volume, RDW: Red cell distribution width, CSF: Cerebrospinal fluid, TNC: Total nucleated cell count.

Laboratory Value	Results	Reference Range
WBC	9.8	4.0-11
HGB	7.9 L	11.3-15.2
HCT	22 L	33-45
Platelets	300	150-400
MCV	83	80-96
RDW	21 H	11.0-16
CSF Glucose	80 H	40-75
CSF Protein	10 L	18-53
CSF RBC	0	0-5
CSF TNC	10 H	0-5
Serum Lead	85 H	0-5
Fecal Lead	28,000 H	N.A
Porphobilinogen	4.3 H	0-1
Zinc Protoporphyrin	218 H	100-200
Urine Protoporphyrin	22 H	0-10
Urine Aminolevulinic Acid	74 H	0-50

**Figure 2:**
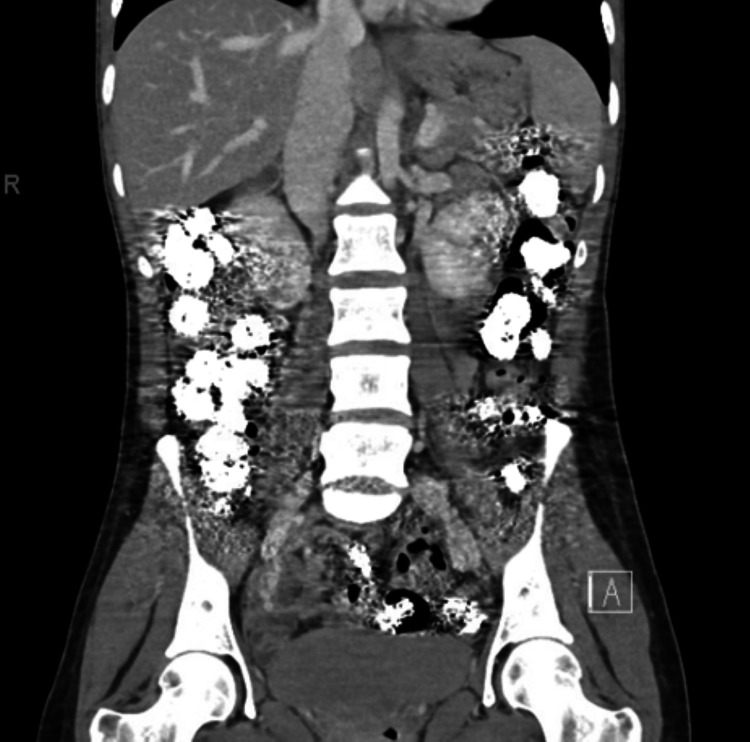
Corneal view CT of the abdomen and pelvis demonstrating hyperdense material

Interestingly, an abdominal plain film and a CT of the abdomen and pelvis displayed scattered hyperdense material within the colonic lumen (Figure 2). Radiology commented that such findings are seen with “recent barium ingestion or calcified stool contents.” The patient also had an abdominal radiograph in the outside hospital records, from three months prior, with a similar radiological interpretation of “residual barium in the colon.” The patient’s family denied knowledge of recent barium procedures leading up to her admission. 

**Figure 3:**
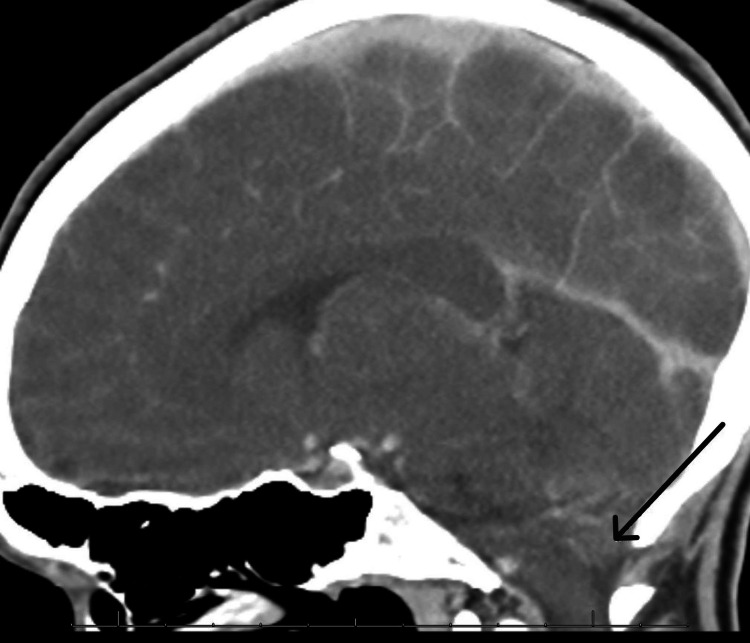
Sagittal CT of the brain after lumbar puncture The image is showing cerebral edema and downward displacement of the brainstem (black arrow).

Twelve hours after the LP (day 3), the patient developed anisocoria with a fixed and dilated right pupil. An emergent head CT scan revealed findings consistent with elevated intracranial pressure (ICP) resulting in downward displacement of the cerebellar tonsils and brainstem concerning for herniation (Figure 3). The patient underwent emergent intubation, extra-ventricular drain (EVD) placement, cerebrospinal fluid (CSF) diversion, and heavy sedation.

Shortly thereafter, the patient’s mother provided new information regarding unknown ‘supplements’ and ‘multivitamins’ provided to the patient by her husband. The husband was not forthcoming regarding supplements. This new history, along with radiopaque findings on radiography, prompted concern for poisoning from heavy metal ingestion. On day five, she was empirically initiated on dimercaptosuccinic acid (DMSA) therapy via nasogastric tube. Calcium disodium ethylenediaminetetraacetic acid (CaNa2EDTA) was considered but unavailable due to national shortage. That evening, a whole blood lead resulted at 85 mcg/dL with a 24-hour urine lead concentration of 4530 mcg/day. A repeat peripheral blood smear demonstrated basophilic stippling. Porphyria testing was abnormal, including elevated free protoporphyrin (22 mcg/dL), urine porphobilinogen (4.3 mcmol/L), zinc protoporphyrin (218 mcg/dL), and urinary aminolevulinic acid (74 nMol/mL). 

**Figure 4:**
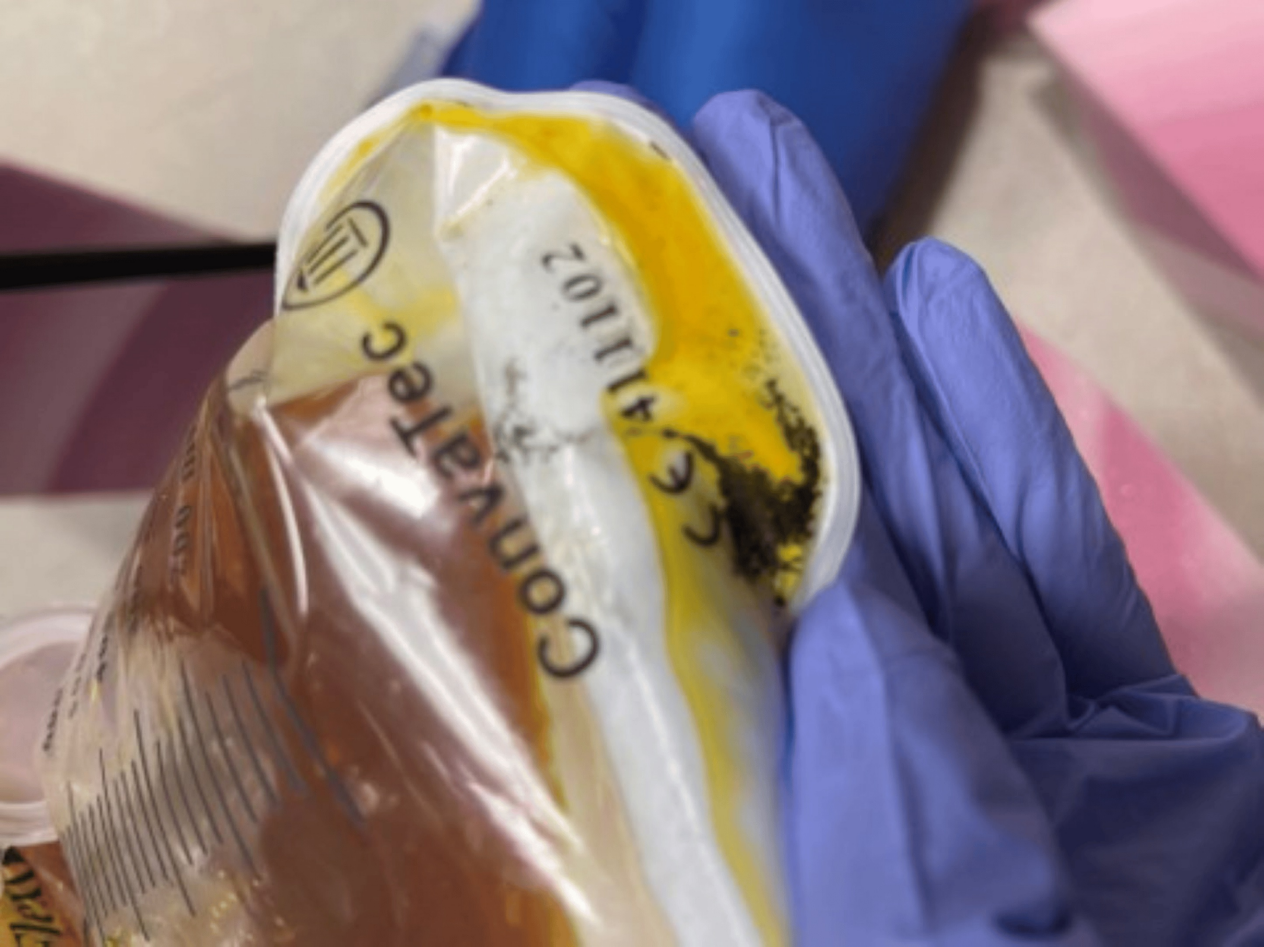
Lead particles in fecal management system

Dimercaprol^TM^ (British Anti-Lewisite: BAL) was subsequently added to treatment on day six. Dual chelation therapy utilizing DMSA 10 mg/kg thrice a day (TID) for five days and then 10 mg/kg twice a day (BID) for 15 days (19 days total), along with BAL (4 mg/kg IM QID) for five days, was completed. Initially, the patient was clinically unstable for endoscopic removal of the lead and instead underwent whole bowel irrigation (WBI). Despite WBI for more than one week, abdominal imaging continued to reveal a persistent large burden of lead in the ileocecal region. A subsequent colonoscopy identified copious lead throughout the lower gastrointestinal (GI) tract (Figure 4) and succeeded in removing the lead burden after failed WBI. Confirmatory testing using mass spectrometry of a stool sample after two days of WBI showed a fecal lead concentration of 28,000 mcg/dL.

After 13 days, the patient’s cerebral edema and herniation improved, permitting EVD removal. She was extubated on day 24 of hospitalization. Her hospital course was complicated by pneumonia and sepsis, pulmonary embolism, and deep venous thrombosis. Renal function remained intact, allowing effective clearance of chelator-metal complexes. The patient underwent tracheostomy (decannulated two weeks after placement) and percutaneous endoscopic gastroscopy tube placement. The patient was discharged on day 43 of hospitalization with outpatient physical therapy. 

**Figure 5:**
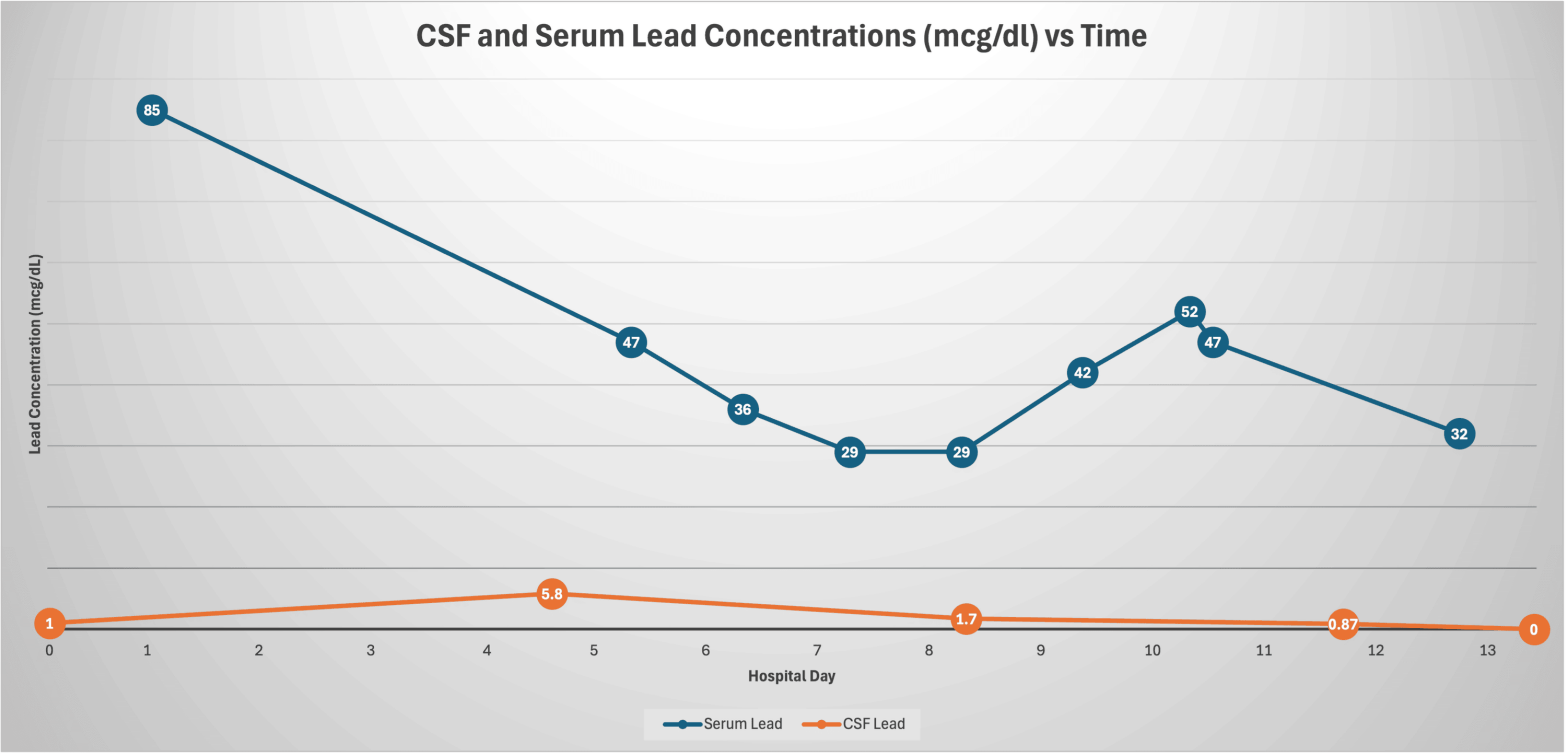
CSF and serum lead concentrations by hospital day

The patient’s initial LP (day 1 of EVD placement) revealed a CSF lead concentration of 1.3 mcg/dL (ref <0.5 mcg/dL), and ICPs ranged from 35-63 mmHg. The subsequent peak was at 5.8 mcg/dL on day 4 (ICPs ranged 10-36 mmHg). On the day of EVD removal (day 13), the CSF lead concentration was 0 mcg/dL and ICPs ranged from 6 to 14 mmHg. Repeat neuroimaging demonstrated improvement of cerebral edema. While undergoing CSF diversion, the patient’s blood lead levels were as follows: Day 1: 85 mcg/dL; Day 4: 47 mcg/dL; Day 12: 32 mcg/dL (Figure 5).

After hospital discharge, the patient was treated two additional times with DMSA, for 19 days each, due to symptomatic rebound of lead concentration with headaches, myalgias, arthralgias, and abdominal pain. At a 10-month follow-up, the patient was back to her baseline functional status with minimal neurological deficits.

All household members were tested for lead, and the patient’s home was evaluated for a source of lead by the state public health department, with no findings. Given the complexity of the social aspects of the case, including the suspected intentional poisoning of the patient by her husband, and at the time, it was unknown if there was any involvement of other family members, a hospital ethics team and legal team consultation was obtained. The ethics and legal team provided guidance on decision-making for the patient and established the ethics team as the patient's next of kin. Additionally, no family members were allowed any contact or visitation rights.

## Discussion

This report highlights the challenges of managing a patient with multiple, non-specific complaints and without an explicit concern for poisoning. Diffuse, severe, intermittent abdominal pain raised suspicion for subacute GI pathology such as porphyria. An unexplained weight loss may be due to malignancy, vitamin deficiency, or paraneoplastic syndromes. Thyroid dysfunction and other endocrinological diseases merited consideration, along with infectious causes such as encephalitis, meningitis, Tropheryma whipplei, and human immunodeficiency virus.

The provided history of naturopathic supplementation sparked a concern for unintentional or intentional toxicity, especially when combined with the unexplained radiopaque findings on abdominal scans. Radiopaque findings raise suspicion for ingestions including barium, iron, chloral hydrate, iodide, other heavy metals, potassium chloride, and paradichlorobenzene [2]. Given subacute encephalopathy and gastrointestinal symptoms, heavy metal treatment was empirically commenced, confirmed by basophilic stippling of a peripheral blood smear. Removal of the colonic lead burden eventually required a colonoscopy; without such a procedure, chelation therapy may paradoxically increase lead absorption from the GI tract.

While cerebral edema and cerebral herniation in adults are typically associated with a very high blood lead concentration (typically >100-150 mcg/dL), these symptoms can manifest at lower concentrations with chronic exposure [1,3]. The patient’s elevated CSF protein concentration, lymphocytic pleocytosis, and MRI findings were also consistent with previous cases of severe plumbism [4-6]. Interestingly, the dangers of lumbar punctures in severe lead encephalopathy have previously been reported in a case series from 1957; like our patient, this report suggests that LP may precipitate cerebral herniation and should be deferred if lead toxicity is strongly suspected [4]. 

To our knowledge, CSF lead concentrations corresponding with ICPs have not previously been reported. The significance of CSF lead concentrations and correlation to ICP is unclear. Stabilization of the patient’s ICP four days after EVD placement, despite a peak in CSF lead concentration, was achieved through diligent ICP management, as discussed in a separate report [7]. Chelation may have paradoxically allowed CSF lead concentration to rise by increasing cerebral distribution of lead from blood, combined with blood-brain barrier dysfunction, as evidenced by malignant cerebral edema.

Treatment of lead encephalopathy begins with cessation of lead exposure [3]. Early initiation of chelation is ideal once encephalopathy presents, with irreversible neuronal damage occurring in at least 25% of survivors [8]. When BAL and CaNa2EDTA are used in combination, mortality is reduced to <5% in lead toxicity [8]. Co-administration of BAL and CaNa2EDTA increases the molar chelator-to-lead ratio, allowing a more efficacious decrease in blood lead concentration; co-administration also mitigates redistribution of lead to the brain [8]. Unfortunately, CaNa2EDTA was in national shortage during this case. Other reports have co-administered DMSA and BAL in lead encephalopathy with success [9,10]. DMSA does not cross the blood-brain barrier, and its beneficial effects are likely secondary to the creation of a concentration gradient between the CNS and the blood [11]. DMSA was used alone in a large cohort of Nigerian children with moderate-to-severe encephalopathy with successful reduction in mortality [12]. However, the long-term morbidity of single chelator use in such severe cases is unknown.

## Conclusions

Without a clear history, the diagnosis of severe adult lead encephalopathy poses several inherent challenges. Symptoms of lead ingestion are vague and can mimic more common disease processes. Clinicians must maintain a high index of suspicion of alternative diagnoses, like lead toxicity, when encountering patients with vague, nonspecific symptoms of headache and abdominal pain. Peripheral smears can be key to earlier diagnosis if basophilic stippling is present. This may be more readily available compared to a serum lead level, which is a send-out test at most institutions. Cases of severe lead toxicity that involve the central nervous system, as in our case, require collaboration among multiple specialists, including emergency department stabilization, GI decontamination, CSF diversion by neurosurgery, and careful ICP management by a neurointensivist. Medical toxicologists play a crucial role in both diagnosis and chelator stewardship. Only through such a cohesive approach was this patient able to have a remarkably successful outcome.
